# Use of Corn Bran with Solubles in Laying Hen’s Diets

**DOI:** 10.3390/ani15152244

**Published:** 2025-07-31

**Authors:** Maria Clara N. Piazza, Ideraldo L. Lima, Ricardo V. Nunes, Kelly M. M. Dias, Romário D. Bernardes, Larissa P. Castro, Beatriz A. Honório, Giovanna L. Vieira, Arele A. Calderano

**Affiliations:** 1 Department of Animal Science, Federal University of Viçosa, Viçosa 36570-900, MG, Brazil; maria.piazza@ufv.br (M.C.N.P.); kelly.maia@ufv.br (K.M.M.D.); duarteromario040@gmail.com (R.D.B.); larissa.p.castro@ufv.br (L.P.C.); beatriz.honorio@ufv.br (B.A.H.); giovanna.vieira@ufv.br (G.L.V.); 2 IL Lima Nutrição Animal, Vila Velha 29101-010, ES, Brazil; ideraldo.lima@gmail.com; 3 Department of Animal Science, State University of Western Paraná, Marechal Cândido Rondon 85960-128, PR, Brazil; nunesrv@hotmail.com

**Keywords:** co-product, egg quality, laying rate, poultry

## Abstract

This study evaluated the production performance and egg quality of Lohmann Brown laying hens fed diets containing different levels of Corn Bran with Solubles (CBS). A total of 144 hens aged 44 weeks were assigned to three treatments in a completely randomized design, with eight replicates per treatment and six birds per replicate. The experimental treatments included diets with CBS inclusion levels of 0%, 5%, and 10%. The experiment lasted 84 days (44 to 55 weeks of age). Data were analyzed via one-way ANOVA, with mean differences evaluated using Tukey’s HSD test (α = 0.05). No significant effects were observed for laying rate, feed intake, feed conversion ratio, or egg mass (*p* > 0.05). However, egg quality parameters such as shell percentage, shell weight per unit surface area (SWUSA), and yolk color were influenced by the treatments (*p* < 0.05). Hens fed diets with 5% CBS exhibited higher shell percentage and SWUSA compared to those on the 0% CBS diet. Yolk color intensity increased with higher CBS inclusion levels. In conclusion, incorporating up to 10% CBS in corn–soybean meal diets for laying hens can enhance egg yolk pigmentation. Notably, including 5% CBS improves eggshell quality.

## Simple Summary

Corn is widely used to produce ethanol, and one of its co-products, called Corn Bran with Solubles (CBS), may be used in animal feed. However, a few studies have evaluated its effects on egg production and quality. This study tested whether CBS could be added to laying hens’ diets without harming their performance. Hens were fed diets with 0, 5, or 10% CBS for 12 weeks, and their production and egg quality were monitored. The results showed that adding CBS did not negatively affect feed intake or egg production. Moreover, a 5% inclusion improved eggshell quality, and both 5 and 10% increased egg yolk color, which is appreciated by consumers. These findings suggest that CBS can be used as a partial replacement for corn in laying hen diets, helping reduce feed costs and promoting the use of alternative ingredients from the biofuel industry. Using co-products like CBS in animal nutrition contributes to more sustainable production systems.

## 1. Introduction

In poultry production, feed represents approximately 70% of production costs and directly influences egg prices. Therefore, continuous efforts are made to develop viable alternative feed sources, such as co-products generated during corn-based ethanol processing. Distillers’ Dried Grains with Solubles (DDGS) is the main co-product studied in poultry nutrition [1,2,3]. The inclusion of corn ethanol co-products in animal diets not only offers a cost-effective strategy but also adds value to the corn ethanol production chain.

To increase productivity in the distilleries, the ethanol sector has increasingly adopted innovative processing technologies. One notable method is the Fiber Separation Technology (FST; ICM Inc., Colwich, KS, USA), which has been implemented by several corn ethanol refineries. This process includes fiber separation as a preliminary step to starch fermentation, improving the efficiency of ethanol production [4]. As a result, two distinct co-products are generated: High-Protein Distillers’ Dried Grains (HP-DDG) and Corn Bran with Solubles (CBS). While the use of HP-DDG in poultry diets has been explored [5,6], limited information is available regarding the nutritional application of CBS.

Recent research has quantified the nitrogen-corrected apparent metabolizable energy and ileal digestibility coefficients of amino acids in CBS [7]. Additionally, average nutritional values for CBS have been published in the Brazilian Tables for Poultry and Swine [8]. Based on this, we hypothesize that CBS can be added at inclusion levels of up to 10% in corn–soybean meal diets for laying hens. Therefore, this study aimed to evaluate the production performance and egg quality of laying hens fed diets containing increasing levels of CBS.

## 2. Materials and Methods

The research was performed at the Federal University of Viçosa, Viçosa, Minas Gerais, Brazil.

At 44 weeks of age, 144 Lohmann Brown laying hens were assigned to three treatments in a completely randomized design, with eight replicates per treatment and six birds per replicate. Allocation to experimental units was based on individual body weight and laying rate, ensuring that the average weight and performance of each unit were as uniform as possible at the beginning of the trial. The hens were housed in metal cages (34 × 47 cm^2^) equipped with trough feeders and nipple drinkers. Each experimental unit consisted of three cages, housing two birds per cage, for a total of six hens per unit.

The experimental treatments consisted of diets containing different inclusion levels of mashed CBS (0%, 5%, and 10%). The CBS used in this study was obtained from a Brazilian ethanol producer (F.S. Bioenergia, Lucas do Rio Verde, Mato Grosso, Brazil) and was manufactured using Fiber Separation Technology (FST; ICM Inc., Colwich, KS, USA). The nutritional profile of the CBS utilized in this study is shown in Table 1.

The experimental diets were based on corn and soybean meals and formulated to meet the nutritional requirements of the hens, according to the recommendations of Rostagno et al. [9] and are presented in Table 2. All diets were provided in mashed form. The metabolizable energy value used for diet formulation was 1849 kcal/kg [7].

During the experimental period, birds had ad libitum access to water and feed, and a 16-h photoperiod was maintained. Ambient maximum and minimum temperatures were monitored daily using two thermometers placed at bird height. The average maximum and minimum temperatures during the experiment were 26.5 °C and 18.4 °C, respectively. The experiment lasted 84 days (44 to 55 weeks of age) and was divided into three data collection periods of 28 days each.

To assess bird performance, the following parameters were measured: laying rate, feed intake (FI), feed conversion ratio per egg mass (FCR), and egg mass. Mortality was monitored and accounted for in the adjustment of performance data. Egg production was recorded to calculate the laying rate daily.

At the end of each 28-day interval, whole eggs were collected over a three-day period to determine average egg weight and egg mass. These eggs were also utilized to evaluate both internal and external quality parameters. Each egg was weighed, opened on a glass surface, and assessed for albumen and yolk quality. The whole egg, shell, and yolk were weighed individually, and albumen weight was determined by calculating the difference between the whole egg weight and the sum of shell and yolk weights. Shell weight was recorded after washing to remove residual albumen and air-drying for 48 h. The percentages of egg components were then calculated. Eggshell thickness was assessed with a digital micrometer at three evenly spaced locations on the equatorial region of the shell, and the arithmetic mean was used as the representative value.

Eggshell density was estimated using shell weight per unit surface area (*SWUSA*) based on the equation proposed by Abdallah et al. [10]:

(1) $$SWUSA=(\frac{SW}{3.9782\;\times \;{EW}^{0.7056}})\;\times \;1000.$$

where *SW* represents shell weight and *EW* represents egg weight, with the result expressed in mg/cm^2^.

The Haugh Unit (*HU*) was determined through the following equation:

(2) $$HU=100\log\;(H+7.57-{1.7W}^{0.37}),$$

where *H* is the albumen height (mm), and *W* is the egg weight (g). Albumen height was recorded using a digital micrometer.

Yolk quality was determined through the yolk index, calculated as the ratio of yolk height to yolk diameter, with both values measured in millimeters. Yolk color intensity was assessed subjectively using the DSM Yolk Color Fan scale (DSM, São Paulo, Brazil).

At 56 weeks of age, blood was collected from two birds per experimental unit via brachial vein puncture. Samples were centrifuged at 3600× *g* for 10 min at 4 °C to obtain the serum, which was subsequently stored at −20 °C until analysis. Serum levels of glucose, triglycerides, and uric acid were determined using a Cobas c 311 analyzer (Roche Diagnostics GmbH, Basel, Switzerland), following the manufacturer’s protocol.

After an 8-h fast, one bird from each experimental unit was slaughtered. Segments of the jejunum (3 cm long) were sampled, washed with a sterile 0.9% sodium chloride solution to eliminate residual luminal content, and opened along the longitudinal axis. The segments were affixed to cardboard with staples, positioning the serosal side against the surface to maintain tissue integrity and avoid deformation. Samples were identified and stored in sterile plastic containers containing 10% buffered formalin for preservation. Following preservation, the samples were dehydrated through a graded ethanol series, cleared in xylene, and embedded in liquid paraffin at 60 °C. Subsequently, the tissue samples were sectioned using a microtome to obtain semi-serial transverse sections of 5 μm thickness, which were subjected to hematoxylin and eosin staining. Each microscope slide received five sections of 5 μm thickness. Slides were analyzed and imaged using an optical microscope (EVOS^®^ XL Core, Thermo Fisher Scientific, Invitrogen, Waltham, MA, USA) at 10× magnification. Villus height (VH), crypt depth (CD), and the VH:CD ratio were quantified with ImageJ software (version 1.50i; Java 1.6.0_20, National Institutes of Health, Bethesda, MD, USA). For each experimental unit, measurements were taken from 20 villi and their associated crypts.

Data were analyzed by analysis of variance (ANOVA) under a completely randomized design using the GLM procedure in SAS software (version 9.4; Statistical Analysis System). The assumptions of residual normality and homogeneity of variances were evaluated using the Shapiro–Wilk and Hartley tests, respectively. Comparisons between treatment means were made using Tukey’s HSD test. A significance level was set at α = 0.05.

## 3. Results

There was no effect of CBS levels in diets on laying rate, FI, FCR, or egg mass (*p* > 0.05; Table 3). Regarding egg quality, the treatments only influenced shell percentage, SWUSA, and yolk color (*p* < 0.05). Eggs from hens receiving a diet with 5% CBS had a higher shell percentage and SWUSA compared to those fed the 0% CBS diet. Egg yolk color increased proportionally with higher CBS inclusion levels. Furthermore, VH, CD, VH:CD ratio, and serum metabolites showed no differences due to the treatments (*p* > 0.05).

## 4. Discussion

In this study, we hypothesized that CBS could be included at levels of up to 10% in corn–soybean meal diets for laying hens. This hypothesis was supported, as CBS inclusion did not negatively affect performance or egg quality parameters. These results were expected, given that CBS is a valuable source of nutrients and energy. Analyses of CBS samples produced in Brazil report average values of 15.6% crude protein, 0.50% total lysine, 8.64% ether extract, and 0.79% phosphorus, along with a nitrogen-corrected apparent metabolizable energy value equivalent to soybean meal [8]. Furthermore, CBS shows high digestibility coefficients for lysine (79.3%) and methionine (91.5%) [7]. Notably, lysine digestibility in CBS is higher than values reported for DDGS in previous studies [6,11,12]. DDGS typically contains lower lysine levels, and its digestibility can vary due to Maillard reactions that occur during thermal processing [13]. In contrast, CBS appears to be less affected during processing. The present results are in line with previous reports suggesting that co-products like CBS can be incorporated into poultry diets at moderate levels without compromising productive performance [4].

A key finding of the present study was the improvement in eggshell quality with CBS inclusion. Relative shell weight and SWUSA improved when 5% CBS was included in the diet. One possible explanation is that the fermentation process could enhance calcium and phosphorus availability among corn ethanol co-products, possibly through the synthesis of microbial phytase [14]. Similar benefits on shell quality have been reported in studies involving DDGS inclusion in laying hen diets [4]. However, the same effect on shell quality was not observed when the diet contained 10% CBS. The higher inclusion of CBS may have limited shell quality responses due to an associated increase in potassium content. Maintaining proper potassium balance is crucial for eggshell formation, as excessive levels may impair nutrient absorption in the gut and, consequently, affect shell development negatively [15].

Another important finding in this study was the increase in egg yolk pigmentation with CBS inclusion in the diets. Visual pigmentation of the yolk is a key attribute of consumer preference for eggs. Since hens are unable to synthesize carotenoids endogenously, the yolk’s carotenoid composition closely reflects that of the diet [16]. Corn, commonly used as the primary component in hen diets, is also the main cereal containing an adequate carotenoid profile for egg yolk pigmentation [17]. The improvement in yolk pigmentation found in this study may be attributed to the greater concentrations of carotenoids in the CBS, which replaced corn and soybean meal in the formulations, as is commonly observed with DDGS [18,19].

This study provides promising evidence supporting the inclusion of CBS as a dietary co-product for laying hens. However, a practical limitation to its broader adoption in commercial feed formulations is its low bulk density, which may increase transportation costs over long distances. One potential solution to this constraint would be to process CBS into pellet form, thereby enhancing its handling properties and transport efficiency.

## 5. Conclusions

CBS can be included in corn–soybean meal diets for laying hens at levels up to 10% without negatively affecting performance, while enhancing yolk pigmentation. Additionally, inclusion at 5% has been shown to improve eggshell quality.

## Figures and Tables

**Table 1:** Composition of Corn Bran with Solubles, as-fed basis.

Dry matter (g/kg)	895.8
Crude protein (g/kg)	183.8
Neutral detergent fiber (g/kg)	393.3
Acid Detergent Fiber (g/kg)	116.8
Crude fiber (g/kg)	101.4
Ether extract by acid hydrolysis (g/kg)	82.8
Ether extract (g/kg)	66.5
Ash (g/kg)	57.9
Potassium (g/kg)	14.4
Total P (g/kg)	11.2
Magnesium (g/kg)	5.06
Sodium (g/kg)	0.98
Calcium (g/kg)	0.21
Iron (mg/kg)	73.3
Zinc (mg/kg)	55.4
Manganese (mg/kg)	18.0
Copper (mg/kg)	4.01
Selenium (mg/kg)	0.17
Gross energy (MJ/kg)	17.45

**Table 2:** Ingredients and nutrient composition of experimental diets (g/kg).

Ingredients	Corn Bran with Solubles		
	0%	5%	10%
Corn	680.61	627.83	575.04
Soybean meal	198.84	189.88	180.93
Limestone	92.70	93.42	94.15
Dicalcium phosphate	12.94	11.93	10.93
Soybean oil	1.57	13.27	24.98
Salt	4.24	4.02	3.79
DL-Methionine, 999 g/kg	3.02	3.14	3.25
L-Lysine HCl, 780 g/kg	1.30	1.47	1.63
Trace mineral premix ^1^	1.10	1.10	1.10
Vitamin premix ^2^	1.00	1.00	1.00
Choline chloride, 600 g/kg	1.00	1.00	1.00
L-Valine, 990 g/kg	0.86	0.97	1.08
L-Threonine, 985 g/kg	0.66	0.78	0.89
L-Tryptophan, 980 g/kg	0.16	0.19	0.23
Corn Bran with Solubles	0.00	50.00	100.0
Calculated Composition			
Metabolizable energy, kcal/kg	2780	2780	2780
Crude protein	148.0	148.0	148.0
Calcium	38.93	38.93	38.93
Available phosphorous	3.18	3.18	3.18
Potassium	5.81	6.21	6.61
Sodium	1.79	1.79	1.79
Digestible lysine	7.36	7.36	7.36
Digestible methionine + cysteine	7.21	7.21	7.21
Digestible valine	6.84	6.84	6.84
Digestible threonine	5.67	5.67	5.67
Digestible tryptophan	1.69	1.69	1.69

^1^ Trace mineral premix provided per kg of product: Mn, 64.20 mg; Zn, 59.63 mg; Fe, 45.85 mg; Cu, 9.14 mg; I, 0.927 mg; Se, 0.275 mg. ^2^ Vitamin premix provided per kg of product: vitamin A, 9637 UI; vitamin D3, 2409 UI; vitamin E, 36.1 UI; vitamin K3, 1.93 mg; vitamin B1, 2.59 mg; vitamin B12, 0.016 mg; vitamin B6, 3.61 mg; vitamin B5, 12.95 mg; vitamin B3, 39.0 mg; vitamin B9, 0.90 mg; biotin, 0.09 mg.

**Table 3:** Effect of different levels of Corn Bran with Solubles in diets on productive performance, egg quality, serum metabolites, and intestinal morphometry.

	Corn Bran with Solubles			SEM	*p*-Value
	0%	5%	10%		
Laying rate (%)	96.16	96.70	95.64	0.32	0.421
Feed intake (g/bird/day)	103.6	105.0	104.3	0.4	0.392
Feed conversion ratio (g/g)	1.83	1.82	1.80	0.01	0.762
Egg mass (g/birds/day)	56.8	57.9	57.9	0.4	0.464
Egg weight (g)	59.1	59.9	60.6	0.4	0.289
Yolk (%)	26.8	26.5	26.2	0.3	0.813
Albumen (%)	63.7	63.7	64.1	0.4	0.863
Shell (%)	9.52 b	9.86 a	9.66 ab	0.05	0.012
Yolk index	0.412	0.409	0.406	0.001	0.331
Haugh Unit	89.86	88.91	89.72	0.49	0.719
SWUSA (mg/cm^2^)	79.49 b	82.67 a	81.29 ab	0.40	0.002
Shell thickness (mm)	0.467	0.471	0.470	0.001	0.100
Yolk color	5.54 c	5.79 b	6.07 a	0.06	<0.001
Glucose (mg/dL)	200.9	199.4	202.3	2.5	0.905
Triglycerides (mg/dL)	1321.6	1319.4	1000.3	66.7	0.070
Uric acid (mg/dL)	2.51	2.46	2.56	0.17	0.977
Villus height (μm; VH)	590.7	674.0	666.0	19.4	0.157
Crypt depth (μm; CD)	158.0	148.8	161.5	5.7	0.666
VH:CD ratio	4.08	4.69	4.17	0.23	0.513

SWUSA: shell weight per unit surface area; SEM: standard error of the means (n = 8 for treatment). Means on the same line, followed by different letters, differ from each other by the Tukey test (*p* < 0.05).

## Data Availability

All relevant data supporting the findings of this study are included in the manuscript.
